# Perception and coding of high-frequency spectral notches: potential implications for sound localization

**DOI:** 10.3389/fnins.2014.00112

**Published:** 2014-05-27

**Authors:** Ana Alves-Pinto, Alan R. Palmer, Enrique A. Lopez-Poveda

**Affiliations:** ^1^Klinikum rechts der Isar, Technische Universität MünchenMunich, Germany; ^2^Medical Research Council Institute of Hearing Research, University ParkNottingham, UK; ^3^Departamento de Cirugía, Facultad de Medicina, Instituto de Neurociencias de Castilla y León, Instituto de Investigación Biomédica de Salamanca, Universidad de SalamancaSalamanca, Spain

**Keywords:** auditory nerve, rate profile, phase-locking, temporal profile, head-related transfer function, HRTF

## Abstract

The interaction of sound waves with the human pinna introduces high-frequency notches (5–10 kHz) in the stimulus spectrum that are thought to be useful for vertical sound localization. A common view is that these notches are encoded as rate profiles in the auditory nerve (AN). Here, we review previously published psychoacoustical evidence in humans and computer-model simulations of inner hair cell responses to noises with and without high-frequency spectral notches that dispute this view. We also present new recordings from guinea pig AN and “ideal observer” analyses of these recordings that suggest that discrimination between noises with and without high-frequency spectral notches is probably based on the information carried in the temporal pattern of AN discharges. The exact nature of the neural code involved remains nevertheless uncertain: computer model simulations suggest that high-frequency spectral notches are encoded in spike timing patterns that may be operant in the 4–7 kHz frequency regime, while “ideal observer” analysis of experimental neural responses suggest that an effective cue for high-frequency spectral discrimination may be based on sampling rates of spike arrivals of AN fibers using non-overlapping time binwidths of between 4 and 9 ms. Neural responses show that sensitivity to high-frequency notches is greatest for fibers with low and medium spontaneous rates than for fibers with high spontaneous rates. Based on this evidence, we conjecture that inter-subject variability at high-frequency spectral notch detection and, consequently, at vertical sound localization may partly reflect individual differences in the available number of functional medium- and low-spontaneous-rate fibers.

## Introduction

The ridges and cavities of the outer ear alter the spectra of sounds that enter the ear canal, mainly (but not only) attenuating energy at high frequencies, such that notches are introduced into the spectra (Shaw and Teranishi, 1968; Lopez-Poveda and Meddis, 1996). These notches are thought useful for judging the vertical location of sound sources (Hebrank and Wright, 1974; Butler and Belendiuk, 1977; Butler and Humanski, 1992; Carlile et al., 2005). In particular, the human pinna introduces a notch whose center frequency increases gradually from around 6.5 to 10 kHz as the vertical location of the sound source moves from −40° to +60° relative to the horizontal plane (for a review see, e.g., Lopez-Poveda, 1996). The bandwidth (BW) of this notch at its 5-dB-down frequencies ranges from ~1 kHz at −40° elevation to ~4 kHz at +10° elevation (Shaw and Teranishi, 1968; Chapter 4 in Lopez-Poveda, 1996). The ability to use these notches for localizing sounds must depend ultimately on the quality of their representation in the auditory nerve (AN), as the nerve is the only path of transmission of acoustic information from the peripheral to the central auditory system^1^. Understanding the nature of the neuronal code underlying the representation of high-frequency spectral notches is therefore pertinent to understanding how sound elevation is perceived. The aim of the present study is to review existing evidence and shed new light on the nature of this code.

The spectrum of a sound may be encoded in the AN activity in at least two ways: in the average discharge rate across fibers tuned to different frequencies (a *rate profile*), and/or in the timing of spikes from fibers tuned to different frequencies. These two mechanisms, however, may not be available for encoding all the temporal and spectral characteristics of a sound. AN fibers can fire in synchrony with a particular phase of the stimulus waveform, a property called “phase-locking,” and this enables them to encode the periodicities of the stimulus waveform in the timing of their spikes. However, as the stimulus frequency increases beyond several kHz, and its period becomes comparable to the variability of synaptic transmission, the jitter of ensuing spike timings degrades the quality of the spectral information. This limits the range of stimulus frequencies that can be encoded in the spiking times of individual fibers (Johnson, 1980; Palmer and Russell, 1986). In other words, this makes the encoding of high-frequency components in the phase-locking of individual fibers ineffective (Delgutte and Kiang, 1984a; Rice et al., 1995; Lopez-Poveda, 2005). Phase locking starts to roll-off at roughly 2 kHz. The frequency beyond which its degradation significantly impacts on spike statistics varies across species, being generally acknowledged to lie at 4 kHz for the guinea-pig (Palmer and Russell, 1986). If a similar 4 kHz phase-locking limit ocurred for humans (and this issue is currently being debated, e.g., Moore and Sek, 2009), then one might presume that the high-frequency spectral notches in the 4–9 kHz range must be encoded via firing rate profiles (Poon and Brugge, 1993; Rice et al., 1995). Here, we present strong evidence that undermines this view.

The question of how high frequency spectral notches are encoded in the AN can be approached by simply testing the hypothesis that they are encoded as AN rate profiles. If this were the case, then the internal, AN representation, and consequently the perception, of high-frequency spectral notches should deteriorate at high sound levels due, firstly, to the broadening of the fibers' frequency response at high levels (Rose et al., 1971), and, secondly, to the saturation of the discharge rate of the majority (~61%) of AN fibers (Rose et al., 1971; Sachs and Abbas, 1974; Evans and Palmer, 1980). While the remaining fibers have wider dynamic ranges (~50–60 dB; Sachs and Abbas, 1974; Evans and Palmer, 1980), only a small proportion of them remain unsaturated at high levels (Palmer and Evans, 1980).

We have previously tested the hypothesis that the internal representation of high-frequency spectral notches deteriorates with increasing sound level in a series of psychoacoustical and computational modeling studies. The results of these studies, reviewed here in the section ‘Human psychophysics’ and ‘Computational simulation of inner hair cell receptor potentials evoked by flat-spectrum and notch noises’ respectively, did not support the rate-profile code and rather pointed to alternative codes. The section ‘Analysis of AN responses to flat-spectrum and notch noises’ presents new data and analyses pertaining to AN activity elicited by stimuli identical to those used in our previous studies. This new set of physiological data also undermines the rate-profile code and rather suggests that the information required for discriminating between noises with different high-frequency spectra is carried in a temporal code. The combined evidence from this series of related psychoacoustical, computational modeling, and physiological studies will be discussed in the last section in terms of its implications for spatial hearing and for the across-listener variability in auditory-based spatial skills.

## Human psychophysics

### Psychoacoustical discrimination between flat-spectrum and notch noises

**Figure 1:**
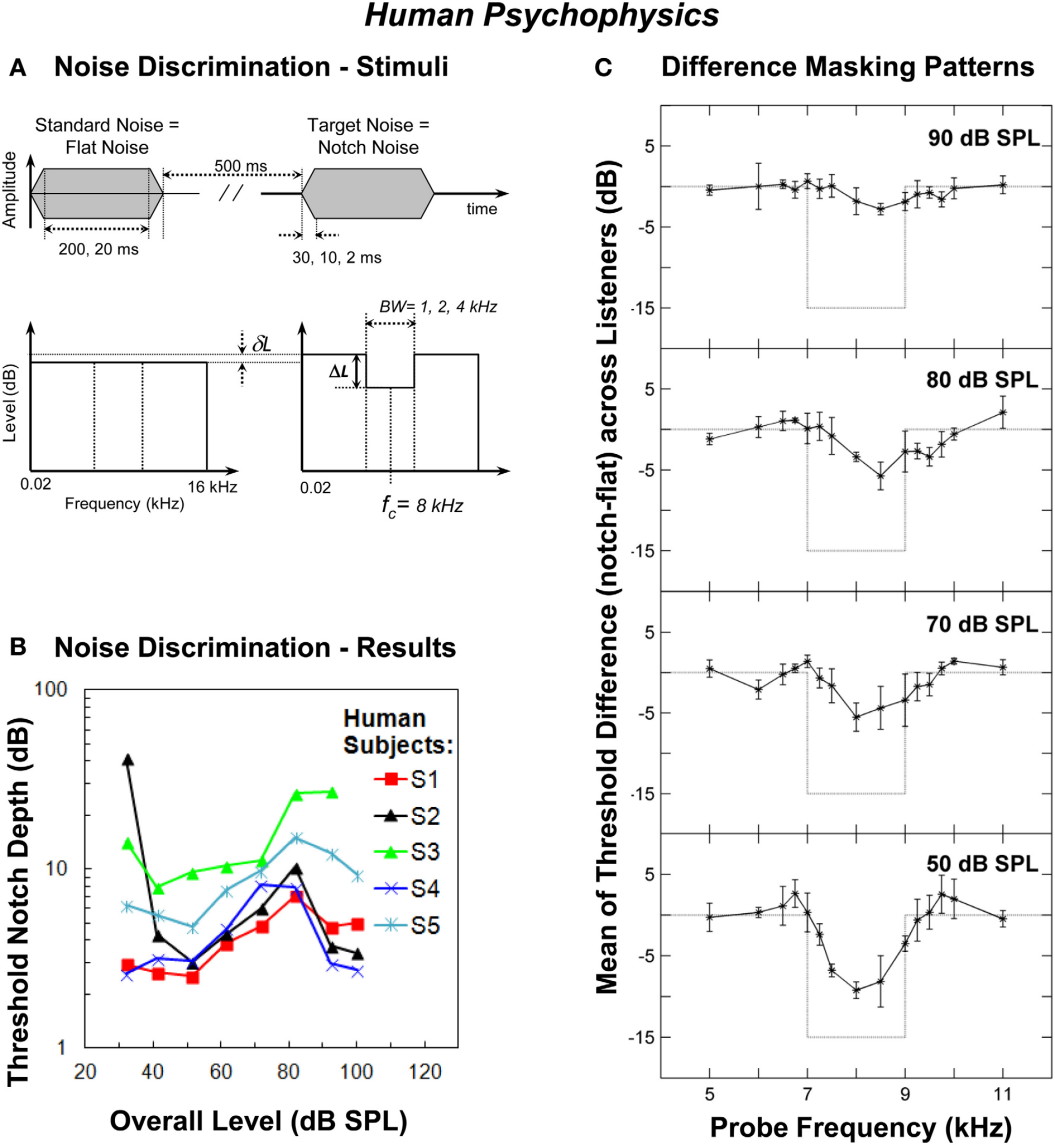
**Human psychophysics. (A)** Schematic description of the waveforms and spectra of the flat-spectrum (“Standard Noise”) and notch (“Target Noise”) noises used in the noise discrimination experiment. The notch depth (Δ*L*) was defined as the difference in dB between the spectrum level in the notch and the reference spectrum level of the noise in the notch side bands. δ*L* represents the reduction in spectrum level applied to the standard noise in order to make its overall level equal to that of the target noise. Also indicated are the values of stimulus duration, stimulus on/off time and the notch bandwidth (BW) tested in the experiments (adapted from Alves-Pinto and Lopez-Poveda, 2005). **(B)** Individual threshold notch depths for discriminating between the standard and target noises (panel **A**) as a function of overall stimulus level. The notch bandwidth was 2 kHz and the notch depth is in dB re the spectrum level in the notch side bands. Each symbol/color illustrates the results for a different listener (adapted from Alves-Pinto and Lopez-Poveda, 2005). **(C)** Differences between the masking patterns of the flat-spectrum and notch noise (notch—flat) at increasing masker levels averaged across listeners. Each panel illustrates the results for a different masker level, as indicated in the top-right corner of the panel. Error bars represent one standard deviation from the mean difference. Dotted lines illustrate the difference between the spectra of the two noises (adapted from Figure 8 of Alves-Pinto and Lopez-Poveda, 2008).

Localization of impulsive sounds in the medial sagittal plane by human listeners deteriorates with increasing sound level up to about 70 dB SPL (Hartmann and Rakerd, 1993). This localization ability is believed to be mediated by the perception of high-frequency spectral notches generated by the filtering action of the human pinna (Hebrank and Wright, 1974; Butler and Belendiuk, 1977; Butler and Humanski, 1992; Carlile et al., 2005). Assuming that the perception of high-frequency spectral features is based on analyzing the AN rate profile then, as with vertical sound localization, the detection of high-frequency spectral notches should become increasingly more difficult as the sound level increases due to the saturation of the fiber firing rates. This hypothesis was tested *psychoacoustically* in humans by measuring the threshold notch depth necessary to discriminate between a flat-spectrum broadband noise and a similar noise with a spectral notch centered at 8 kHz (Figure 1A) at increasing noise levels, from 32 to 100 dB SPL (Alves-Pinto and Lopez-Poveda, 2005). If the hypothesis were true, then notch detection thresholds should increase, i.e., discrimination should become increasingly more difficult, with increasing noise level, as a result of the deterioration of the AN rate-profile representation of the spectral notch at high levels.

Surprisingly, however, threshold notch depth varied *non*-monotonically with level for most, but not all, listeners, increasing up to about 70–80 dB SPL and decreasing for higher levels (Figure 1B). The non-monotonic effect, when present, was comparable for notch BWs of 1, 2, and 4 kHz (see Figure 6 of Alves-Pinto and Lopez-Poveda, 2005), and for stimulus durations of 20 and 200 ms (see Figure 8 of Alves-Pinto and Lopez-Poveda, 2005), even though notch depth thresholds were generally higher for narrower notches and shorter stimuli. Stimulus rise times (2, 10, or 30 ms) did not affect notch depth thresholds at any of the levels tested (see Figure 7 of Alves-Pinto and Lopez-Poveda, 2005). These observations suggest that the non-monotonic shape of the threshold notch depth vs. level function is independent of stimulus duration and of the number of AN fibers that “see” a difference in energy between the two stimuli, that is, the fibers' with CFs within the notch frequency band.

Hence, the initial hypothesis of a monotonic increase in notch detection thresholds with increasing level was not supported by the experimental results, which rather suggested that the notch must be better represented internally at levels above and below around 70–80 dB SPL than at these mid-levels. This result prompted further research aimed at investigating the quality of the internal representation of the spectra of flat-spectrum and notch noises at increasing sound levels using diverse approaches: first, by comparing psychoacoustical masking patterns evoked by the two noises; second, by comparing computer simulations of the peripheral auditory system response to the two noises; and lastly, by analyses of direct AN fiber responses to the two noises.

### Psychoacoustical masking-pattern representation of high-frequency spectral notches

The quality of the rate-profile representation of flat-spectrum and notch noises was assessed psychoacoustically by measuring the forward-masking patterns of the two noises (Alves-Pinto and Lopez-Poveda, 2008). A masking pattern is a graphical representation of the detection thresholds of masked probe tones as a function of probe frequency. Psychoacoustical forward masking is thought to reflect (to a large extent) the incomplete recovery of AN fibers from previous stimulation and/or the persistence of neural (post-AN) activity (Oxenham, 2001; Meddis and O'Mard, 2005). Whatever the case, detection of a low-level tonal probe is likely mediated by the *average* discharge rate evoked by the probe in AN fibers with CFs similar to the frequency of the probe. When the probe is preceded by a masker sound, this rate almost certainly changes depending on the activity evoked by the masker in those same fibers (Harris and Dallos, 1979; Meddis and O'Mard, 2005). Hence, the activity evoked by the flat-spectrum noise on AN fibers with CFs within the notch band would be likely different from that evoked by the notch noise. This difference should be reflected as a difference in masked probe detection thresholds and, consequently, in the masking patterns produced by the two noises. Furthermore, by presenting the probe after the masker any potential interactions between the two stimuli (e.g., suppression, distortion, or beating effects) are minimized, thus favoring forward masking to psychoacoustically assess the quality of the internal representation of the two noises.

The forward masking pattern of the flat-spectrum/notch noises were obtained by measuring the masked threshold of detection of pure tones with frequencies covering the spectral region of the notch. They were measured for low (50 dB SPL), medium (70 and 80 dB SPL), and high (90 dB SPL) masker overall levels, to allow comparison with the non-monotonic effect of level in the main discrimination task (Figure 1B). The quality of the internal representation of the spectral notch was inferred from the difference between the masking patterns of the flat-spectrum and notch noises.

The spectral notch was clearly visible in the difference masking patterns at 50 dB SPL, less obvious at 70 and 80 dB SPL, and barely visible at 90 dB SPL (Figure 1C). The fact that the two masking patterns became more similar as the level increased from 50 to 80 dB SPL is consistent with the increase in discrimination threshold notch depth over the same level range (Figure 1B). Above 80 dB SPL, however, the difference between the two masking patterns continued to decrease (Figure 1C, upper panel) even though notch detection became easier (i.e., threshold notch depth generally decreased above around 80 dB SPL, Figure 1B). Insofar as a masking pattern is regarded as the psychoacoustical correlate of a neural excitation pattern, this result suggests that discrimination between the flat-spectrum and notch noises is, at least above 80 dB SPL, unlikely based on comparisons of the AN rate-profile representations of the noise spectra.

## Computational simulation of inner hair cell receptor potentials evoked by flat-spectrum and notch noises

The quality of the internal AN representation of high-frequency spectral notches must be limited by the signal processing that takes place before the AN. The inner hair cell (IHC) receptor potential is the driving potential of AN fibers' activity and therefore sets a limit on the quality of the representation of spectral information in the AN. It is possible, for example, that the excitation pattern representation of the stimulus spectrum degrades at high sound levels because saturation already occurs at the level of the IHC receptor potential (e.g., Russell and Sellick, 1978). For this reason, the quality of the representation of high-frequency spectral notches was assessed pre-AN by using a computational model of *receptor potential signals* generated by a bank of IHCs in response to flat-spectrum and notch noises (Lopez-Poveda et al., 2008). Assessing the quality of the representation high-frequency notches at the level of the receptor potential is advantageous also because the receptor potential is a deterministic, continuous signal that is easier to analyze than stochastic, discrete signals like AN spike trains.

**Figure 2:**
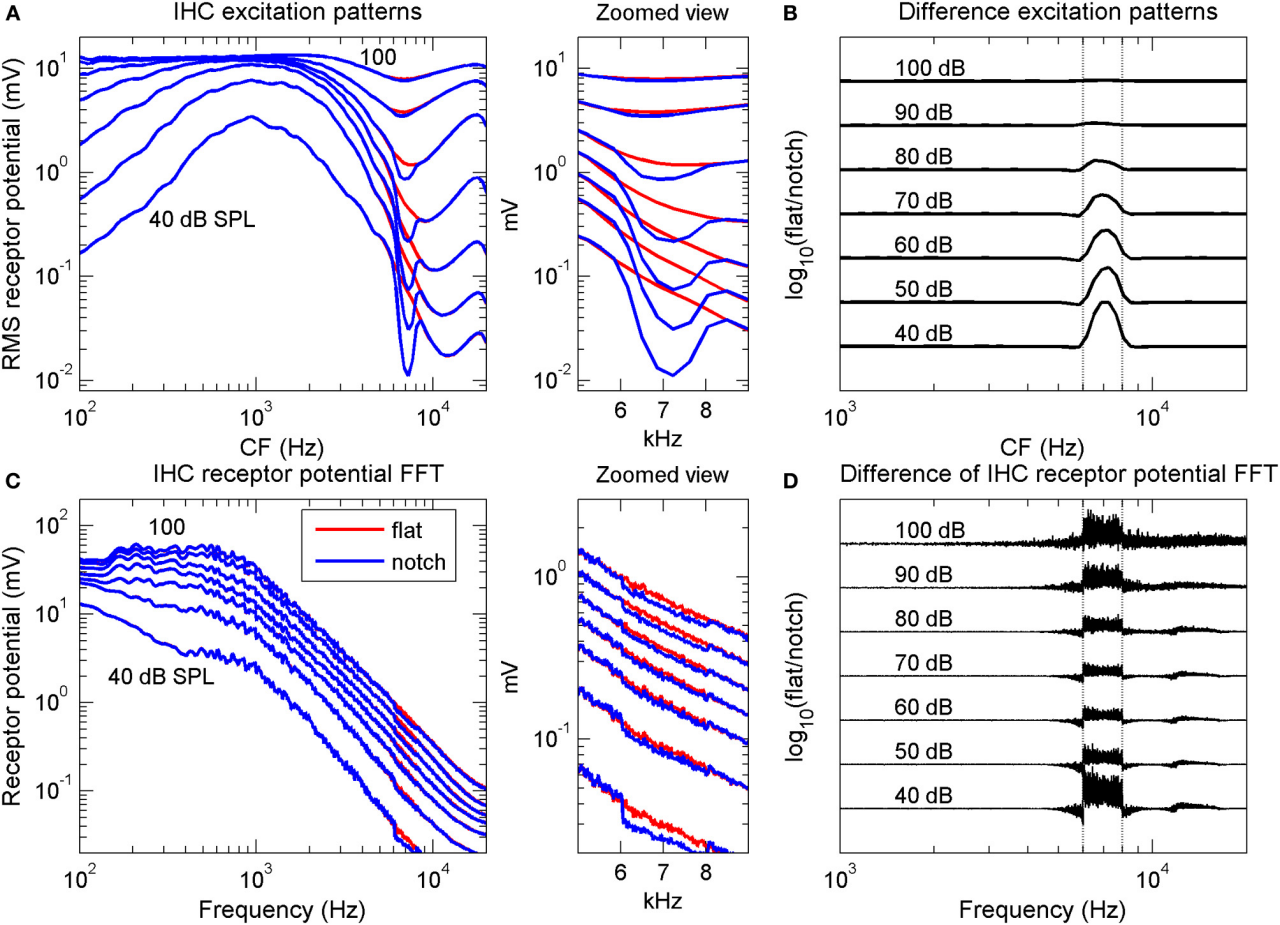
**Computational simulation of inner hair cell receptor potentials**. Simulated IHC responses to broadband noises with a flat spectrum and with a 2-kHz wide, 15-dB spectral notch centered at 7 kHz. The noise duration was longer than that used in the psychoacoustical experiments (0.5 vs. 0.2 s) to obtain “smoother” responses. **(A)** IHC excitation pattern representation of the flat-spectrum (red) and notch noises (blue). Each curve illustrates the average (rms) receptor potential of each IHC as a function of the cell's CF, for a different stimulus level, from 40 to 100 dB SPL, as indicated by the numbers next to each trace. **(B)** Difference excitation patterns (in dB) normalized to the maximum value across CFs and intensities. The numbers next to each trace indicate stimulus intensity in dB SPL. **(C)** Spectra of the IHC receptor potential representation of the two noises for the same stimulus levels as in **(A)**. Each curve depicts the frequency-wise summed spectra of individual IHC receptor potential spectra (see main text). **(D)** Difference receptor potential FFT (in dB) normalized to the maximum value across frequencies and intensities. In **(B,D)**, the curves have been arbitrarily displaced vertically for convenience. Vertical dotted lines in **(B,D)** indicate the notch frequency band. The middle panels illustrate zoomed views of panels **(A,C)** over the frequency range of the spectral notch.

The model included realistic cochlear mechanical level-dependent gain and tuning and a realistic IHC model (see Lopez-Poveda et al., 2008 for details). The model was evaluated in the time domain in response to both a flat-spectrum broadband noise and a noise with a 15-dB deep, 2-kHz wide, rectangular spectral notch centered at 7 kHz. The levels of the two noises were identical to those used in the psychoacoustical spectral discrimination task. The model output was a collection of receptor potential waveforms for a bank of IHCs with different CFs. The receptor potential waveforms were analyzed in two different ways: first, by plotting the root-mean-square (rms) receptor potential amplitude of each IHC as a function of the cell's CF—an *excitation pattern* representation (Figure 2A). This representation is akin to the AN rate profile representation of the stimulus spectrum, since the average discharge rate of an AN fiber is thought to be proportional to the rms receptor potential of its corresponding IHC (Cheatham and Dallos, 2001). The second analysis method involved: (1) applying a fast Fourier transform (FFT) to the receptor potential waveform of each IHC in the bank; and (2) adding all the resulting spectra, one per IHC, in the frequency domain to obtain a *population receptor potential FFT* representation of the stimulus spectrum (Figure 2C). This population response spectrum roughly reflects the total magnitude of phase-correlated response of the whole IHC population. In the real ear, each IHC would be actually innervated by several AN fibers, all of which would be driven by a common IHC receptor potential waveform. The FFT of an individual IHC receptor potential waveform represents an upper boundary to the temporal periodicities that could be encoded by the group of AN fibers innervating that IHC in their aggregated spike times. Likewise, the aggregated receptor potential FFTs for all IHCs represent an upper boundary to the periodicities that could be encoded by the population AN, hence providing a representation akin to the phase-locking representations in the AN (further details in Lopez-Poveda et al., 2008).

The results of the simulations showed that the quality of the IHC excitation pattern representation of the spectral notch (blue line in Figure 2A) degraded gradually with increasing stimulus intensity, a result clearly visible in the difference excitation patterns (Figure 2B). Differences between the two excitation patterns occurred for IHCs with CFs within or around the notch band only, with the largest difference occurring for the lowest intensity (40 dB SPL). By contrast, differences in the simulated IHC population receptor potential FFTs were smaller at mid intensities, around 60–80 dB SPL, than at lower and higher intensities (Figures 2C,D). Interestingly, significant differences occurred for frequencies outside the notch frequency band, particularly at the highest intensities (Figure 2D).

If psychoacoustical discrimination between the flat-spectrum and notch noise were determined by differences between the IHC representations of the flat-spectrum and notch noise spectra, then the simulations suggested that discrimination based on the excitation pattern should be increasingly more difficult with increasing level (Figure 2B), whilst discrimination based on the population receptor potential FFT should be easier below and above 70 dB SPL (Figure 2D). Only the latter is *qualitatively* consistent with the non-monotonic shape of the psychoacoustical threshold notch depth *vs*. level functions (Figure 1B).

What is the origin of the non-monotonic effect of level in the population receptor potential FFT? This issue was addressed by Lopez-Poveda et al. (2008). In short, they suggested that the gradual decrease in notch sensitivity up to 60–80 dB PSL is due to the cochlear mechanical compression whilst the improvement at high levels seemed to be due to IHC nonlinearities: at high sound levels, the flat-spectrum noise saturates the population IHC receptor potential more than does the notch noise and this would alter the spike patterns of AN fibers innervating a saturated IHC relative to those innervating a non-saturated IHC (see Lopez-Poveda et al., 2008 for a detailed explanation).

Even though the model may not perfectly simulate the human IHC response (Lopez-Poveda et al., 2008), the simulations suggested two important aspects about the nature of the code underlying the psychoacoustical discrimination between flat-spectrum and notch noises. First, that the quality of the IHC excitation pattern representation of the spectral notch decreased gradually with increasing sound level (Figure 2B) means that the quality of the AN rate profile must necessarily decrease with increasing intensity, regardless of the type of AN fiber. This undermines the suggestion that the peak in the behavioral threshold notch depth *vs*. level function (Figure 1B) reflects the transition between the dynamic ranges of AN fibers with low and high thresholds, according to which the notch would be encoded in the activity of low-threshold (or high-spontaneous rate, HSR) fibers at low to mid-levels and on that of high-threshold (or low-spontaneous rate, LSR) fibers at high noise levels (Alves-Pinto et al., 2005).

Second, the similarity between the effects of intensity on the difference IHC receptor potential FFT (Figure 2D) and the threshold notch depths for spectral discrimination (Figure 1B) suggests that high-frequency spectral discrimination could be based on comparisons of internal representations of the spectra obtained by precise analysis of the *timing* of AN spikes. The actual mechanism that would allow the central auditory system to extract such a representation is uncertain (see below), but the model simulations suggested that it could be similar in effect to a Fourier transform of the spike trains (Young and Sachs, 1979). This would imply that useful frequency information is actually encoded in the timing of AN discharges even at stimulus frequencies at which phase-locking is significantly diminished (>4 kHz; Palmer and Russell, 1986). A similar conjecture has been put forward by a modeling study on the limits of human auditory perception of single tones (Heinz et al., 2001). Heinz et al. suggested that psychoacoustical frequency difference limens are consistent with frequency information being encoded in the discharge times of AN fibers for frequencies up to 10 kHz. This has been supported by recent physiological studies that have shown that detectable phase-locking can occur for frequencies as high as 14 kHz (Recio-Spinoso et al., 2005).

Inspired by this, further insight about the neuronal code responsible for the internal representation of high-frequency spectral notches and for the main psychoacoustical discrimination results was sought by directly measuring the activity of AN fibers in response to the flat-spectrum and notch noises used in the psychoacoustical and simulation experiments. These new data are described in the following section.

## Analysis of auditory nerve responses to flat-spectrum and notch noises

### Rationale

The quality of the internal representation of the high-frequency spectral notch at the level of the AN was assessed physiologically by directly recording the activity of guinea-pig AN fibers in response to stimuli like those used in the main psychoacoustical study. Following the evidence from the psychoacoustical and simulation studies (reviewed above), analyses of neuronal activity included an evaluation of the representation of the spectral notch in the average rate profile, but also in the temporal pattern of ANfiber discharges. For the latter, we could not apply the FFT analysis that we had used to analyze IHC receptor potential simulations because of (1) the discrete nature of the AN spike trains, (2) the short duration of the recording interval (110 ms), and (3) the limited number of recorded AN units. Instead, we used an “ideal observer” analysis (see below).

### Methods

#### Physiological recordings

Recordings from AN fibers of anaesthetized guinea pig were made using the methods described in Palmer et al. (1986). Data were collected from 163 fibers (from 18 animals) with CFs between 0.9 and 19 kHz, a CF range sufficient to cover the relevant spectral content of the stimulus. Fifty three of the 163 fibers had spontaneous rates less than 18 spikes/s, i.e., had low-to-medium spontaneous rates, a proportion consistent with the distribution of the different types of fibers in the guinea pig in terms of spontaneous rate and threshold levels (Yates, 1991).

#### Stimuli

AN fibers were stimulated with bursts of broadband (0.02–16 kHz) noise similar to those used in the psychoacoustical and simulation experiments. Two types of noises were used: one had a flat spectrum; the other was similar except for a frequency region centered at 7 kHz where it had a rectangular spectral notch (Figure 1A). The spectrum level in the notch band was 0 (i.e., flat spectrum), 3, 6, 9, 15, 21, or 27 dB below the spectrum level outside the notch band. Notch BWs of 2 and 4 kHz were used. Stimuli were presented for overall levels ranging from 40 to 100 dB SPL in 10-dB steps. Noise bursts had a total duration of 110 ms, including a 10-ms rise time; no fall ramp was applied. A different stimulus condition, defined by the notch depth and the overall sound level of the stimulus, was presented every 880 ms. Conditions were presented in random order.

The noise bursts were generated as described in the related behavioral study (Alves-Pinto and Lopez-Poveda, 2005). A single noise token was generated in the digital domain for each notch depth and used for repeated measures of AN responses at all levels (i.e., the noise was “frozen”). The noise bursts used in the present study were shorter (110 ms *vs*. 220 ms) and the notch center frequency was lower (7 kHz *vs*. 8 kHz) than those used in the related psychoacoustical study. Despite these differences, the fundamental characteristics of the stimuli remained the same: in both cases the notch frequency band was beyond the cut-off frequency of phase-locking (~4 kHz according to Palmer and Russell, 1986), and the stimulus duration was longer than the fast-adaptation period of AN fibers (~30 ms according to Westerman and Smith, 1984).

#### Rate profile analysis of auditory nerve responses

In this analysis a subpopulation of 106 fibers, for which at least 5 and typically 10 complete spike trains were recorded for all stimulus conditions tested, was used. The mean discharge rate was calculated over the whole stimulus duration (110 ms). Raw rate profiles are uninformative of the spectral content of the stimulus due to the large across-fiber variability in spontaneous and saturated rates (Rice et al., 1995). To account for the rate variability across fibers, normalized rate profiles (varying from 0 to 1) were used instead. The normalization was done as follows (Rice et al., 1995): *R*_norm_ = (*R* – *SR*)/(*R*_max_– *SR*), where *R* is the average discharge rate of the fiber, *SR* its spontaneous rate, and *R*_max_ its maximum discharge rate. Here, *SR* and *R*_max_ were estimated as the average discharge rates for a flat-spectrum noise stimulus of 40 and 100 dB SPL, respectively. Due to the small number of fibers with low-to-medium spontaneous rates (31 fibers only), reliable rate profiles for separate fiber type groups could not be obtained. Instead, the whole unit sample was used to properly sample the frequency range of interest in a rate profile. In the related behavioral task (Psychoacoustical discrimination between flat-spectrum and notch noises), subjects were asked to discriminate between a flat-spectrum noise and a noise with a spectral notch. Therefore, difference rate profiles for the two stimuli were also calculated as they provide a more relevant neural correlate of psychoacoustical performance than do normalized rate profiles. All rate profiles were smoothed by applying a running average calculated over 1/3rd-octave-band intervals.

#### “Ideal observer” analysis of auditory nerve responses

The psychoacoustical threshold notch depth for discriminating between a flat-spectrum and a notch noise, Δα, was predicted from the responses collected for the sample of AN fibers according to the following equation (Siebert, 1970; Heinz et al., 2001):

(1) $$\Delta \alpha ={\left\{\sum_{i}\int_{0}^{T}\frac{1}{r_{i}\left(t,\alpha \right)}{\left[\frac{\partial r_{i}\left(t,\alpha \right)}{\partial \alpha }\right]}^{2}dt\right\}}^{-0.5},$$

where *t* denotes time, *T* denotes the stimulus duration, and *r*_*i*_(*t*, α) the instantaneous discharge rate of the *i*-th fiber in response to the stimulus with notch depth α. The term in square brackets determines the change in instantaneous discharge rate *r*_*i*_(*t*, α) of the *i*-th fiber at a given time instant, *t*, as a result of a change, ∂α, in the stimulus condition. This term is squared to make positive and negative changes equally relevant. This change is then divided by the fiber's “instantaneous” discharge rate *r*_*i*_(*t*, α), a sort of “normalization” procedure that takes into account the fiber's particular physiological characteristics. This is important because, for example, whilst a change of 1 spike/s may be meaningless for an HSR fiber, it may represent a huge change for a LSR fiber whose average discharge rate can be below 1 spike/s. The relative change in discharge rate is summed [integral in Equation (1)] throughout the stimulus duration, *T*, providing a measure of the overall sensitivity of this *i*-th fiber to a change ∂α in the stimulus. These individual sensitivities are then summed across fibers to obtain a measure of the ability of the *sample* of fibers to indicate a change in the stimulus conditions through a change in discharge rate of any of the fibers.

Given the discrete nature of the recorded AN responses and the limited number of stimulus conditions tested, a discrete version of the above equation was adopted for the current analysis:

(2a) $$\Delta \alpha ={\left\{\sum_{i}\sum_{k\;=\;1}^{nbins}s_{i,k}\right\}}^{-0.5}$$

where *s_i,k_* is the *sensitivity* of the *i*-th fiber over the *k*-th time bin and is defined as follows:

(2b) $$s_{i,k}=\frac{1}{r_{i}\left(\Delta t_{k},0\right)}\cdot {\left[\frac{r_{i}\left(\Delta t_{k},0\right)-r_{i}\left(\Delta t_{k},3\right)}{3-0}\right]}^{2}\cdot \Delta t_{k}$$

where *k* is the index for the time bins considered in the analysis. The “instantaneous” discharge rate is replaced in Equation (2b) by the average discharge rate within a time interval (time bin) of duration Δ*t*. *r*_*i*_(Δ*t_k_*, 0) is then the average discharge rate in the *k*-th time bin in response to the flat-spectrum noise (notch depth = 0 dB), and *r*_*i*_(*t_k_*, 3) the average discharge rate in response to the 3-dB-deep notch noise. This was the smallest notch depth for which AN responses were recorded, and so it was assumed analogous to the incremental change ∂α of the stimulus parameter in Equation (1). Hence, the relative change in average discharge rate in each time bin, between responses to flat-spectrum and 3-dB-deep notch noises, was calculated for each fiber and added across time bins and across fibers.

**Figure 3:**
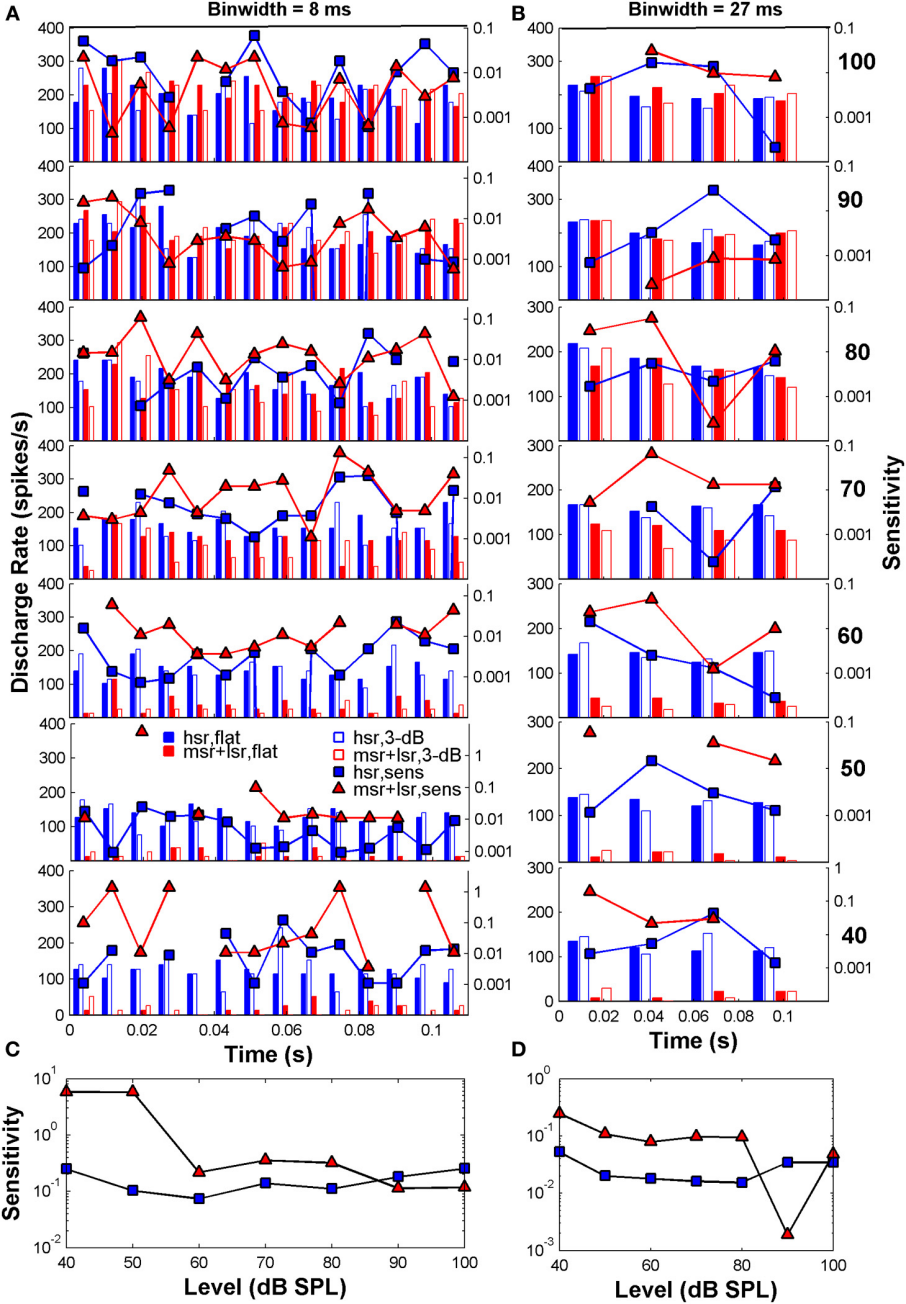
**Auditory nerve data: example post-stimulus time histograms (PSTHs; scale on the left y-axis) and related sensitivity (scale on the right y-axis) for one HSR fiber (blue bars and squares: CF = 3.6 Hz, SR = 111 spikes/s, 10 repeats/stimulus) and one LSR fiber (red bars and triangles: CF = 6.9 Hz, SR = 11.2 spikes/s, 10 repeats/stimulus). (A)** PSTHs calculated for time binwidths of 8 ms. **(B)** PSTHs calculated for a binwidth of 27 ms. In each panel, filled and open blue bars illustrate the PSTHs for the HSR fiber when stimulated with a flat-spectrum and 3-dB-deep notch noise, respectively. Filled and open red bars illustrate corresponding PSTHs for the LSR fiber. Each row illustrates results for a different stimulus level as indicated by the bold numbers on the right part of the figure (in dB SPL). Also represented in each panel is the fiber's sensitivity in each time bin (log-scale on the right y-axis) for each of the two fibers (blue squares for the HSR fiber; red triangles for the LSR fiber; one symbol per bin). Sensitivity was calculated using Equation (2b) and yields a measure of a fiber's ability to discriminate between the two stimuli through a change in the discharge rate evoked by them, in different time bins. Missing symbols indicate bins for which the two stimuli elicited identical discharge rates, hence sensitivity became zero. **(C)** Overall sensitivity as a function of stimulus level for each of the fibers represented in panel **(A)**. Overall sensitivity for a given level was obtained by summing all the sensitivities across all bins for that level [Equation (2a)], represented by the symbols in the corresponding panel **(A)**. Blue squares and red triangles illustrate the sensitivity *vs*. level function for the HSR and LSR fibers, respectively. **(D)** The same as in C but for time binwidths of 27 ms. Overall sensitivity for each fiber was obtained by summing all the sensitivities at the corresponding level in panel **(B)**. The results presented in all panels are based on the responses of the same two AN fibers. For each fiber, different sensitivities within each time bin (panels **A,B**) produce different sensitivity *vs*. level functions (panels **C,D**).

Figure 3 illustrates example post-stimulus time histograms elicited by the flat-spectrum noise (filled bars) and the 3-dB notch noise (open bars) for two *individual* fibers: an HSR fiber (blue bars; CF = 3.6 Hz) and an LSR fiber (red bars; CF = 6.9 Hz) fiber. The discharge rate scale is on the left y-axis. Each stimulus typically elicits different discharge rates in each time bin (Figure 3A). This difference in discharge rate is the basis for the sensitivity of that single fiber to the two different stimuli. The sensitivity in each time bin was calculated using Equation (2b) and is represented by the blue squares (HSR) and red triangles (LSR) in Figures 3A,B (referred to the log-scale on the right y-axis). When similar discharge rates are evoked by the two stimuli the fiber is unable to distinguish between the two simply based on the rate difference information, and consequently its sensitivity becomes zero (missing symbols in some bins in Figure 3A). Summation of all these sensitivities across bins yields an overall measure of sensitivity at a given level for that individual fiber and consequently to an individual sensitivity (or its inverse, a discrimination threshold estimate) *vs*. level function for that fiber (Figures 3C,D). The sensitivity also depends on the binwidth [Equation (2b)]. Assessing the discharge rate using longer time bins (Figure 3B; responses are for the same two fibers represented in Figure 3A, only the binwidth for computation of the discharge rate is different) produces different patterns of discharge and consequently produces different sensitivities and discrimination thresholds (Figures 3C,D; notice the different scales in the right y-axis).

It becomes evident that this analysis is designed to detect the maximum relative change in discharge rate available throughout the stimulus duration and throughout the population of fibers and that it optimizes the information that each fiber can convey in its response toward the detection of a change in the stimulus, hence the term “ideal observer” analysis. The information carried in the variance of firing rate in each time bin counts and, in this sense, this “ideal observer” analysis contrasts with the average rate profile analysis that disregards any rate fluctuations in time and considers only the information conveyed in the overall discharge rate of the fibers assessed throughout the whole stimulus duration.

Equation (1) was derived on assumption that the occurrence of AN spikes follows a Poisson distribution, that is, that spikes occur at times that are independent of each other. Furthermore, in using Equation (1) to predict psychoacoustical discrimination thresholds, the implicit assumption is made that the listener can make optimal use of every bit of information available in the activity of the population of fibers, as explained above. Although neither of these two assumptions apply here (Siebert, 1965, 1968, 1970), we assumed that the error in using Equation (2) for predicting the psychoacoustical thresholds is comparable for all sound levels, and hence that Equation (2) serves to qualitatively predict how threshold notch depths change with sound level, as reported in the related psychoacoustical study (Alves-Pinto and Lopez-Poveda, 2005).

Δα was computed for different time bin durations, Δ*t*, from 0.333 to 110 ms. For Δ*t*s that were not submultiples of the stimulus duration, the last bin, that had a different duration from the other bins, was eliminated from the sum in Equation (2a). Eliminated bins were no longer than 2 ms. When Δ*t* is set to the stimulus duration, the resulting Δα corresponds to performance based on a rate-profile code only. Δα becomes unrealistically equal to zero when the discharge rate of any fiber is equal to zero for any bin (no bar for the 3-dB notch noise at some of the time bins in Figure 3A). To prevent this artifactual result, a small, arbitrary constant of 0.1 spikes/s was added to the measured discharge rate in all bins of all fibers. The actual value of this constant did not alter results significantly. The results presented are based on the results of the group of 163 fibers for which at least 5 and typically 10 repeats were recorded for a flat-spectrum noise and for a notch depth of 3 dB at each of the different sound levels tested.

### Results

#### An rate profiles do not explain psychoacoustical noise discrimination as a function of level

**Figure 4:**
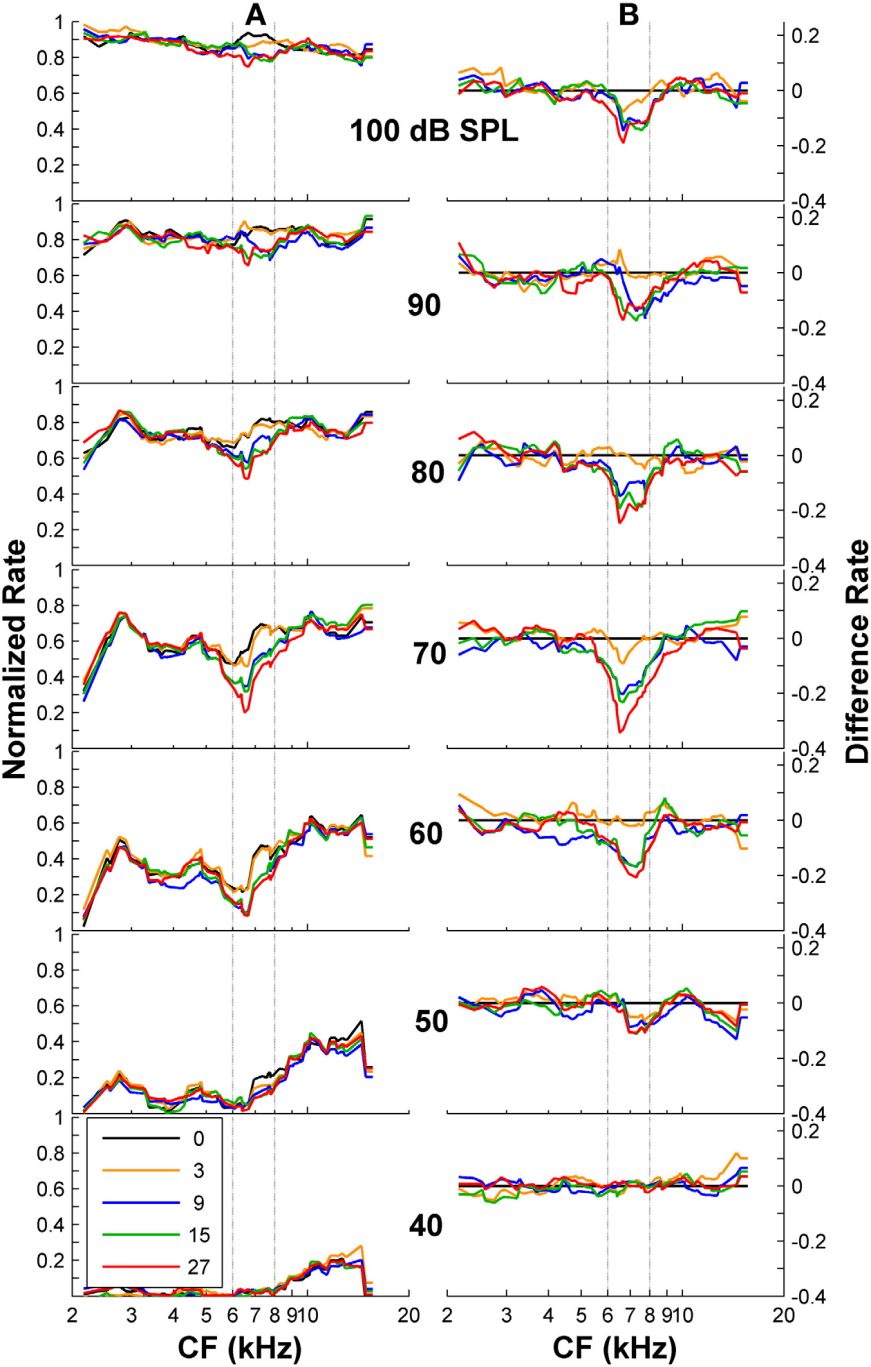
**Auditory nerve data: rate profiles for different overall noise levels and for different notch depths. (A)** Normalized rate profiles. Each curve is for a different notch depth (in dB), as indicated by the inset. **(B)** Difference between the rate profiles for the flat-spectrum and the notched noises. The numbers in the inset denote notch depths in dB re spectrum level of the notch side bands. Vertical dashed lines illustrate the frequency band of the spectral notch.

First, we tested whether psychoacoustical spectral discrimination could be accounted for using only the AN rate-profile representation of the stimulus spectrum. A simple visual analysis of both normalized and difference rate profiles (Figures 4A,B) revealed a lower discharge rate for those fibers with CFs around the frequency band of the notch, with deeper notches eliciting lower discharge rates at mid-levels. This would suggest that AN rate-profile comparisons constitute a reasonable physiological basis for psychoacoustical discrimination of high-frequency spectra. However, a closer look disproves this suggestion: the absolute rate difference was largest for overall levels around 60–80 dB SPL. This implies that discrimination should be easiest around these levels, in clear contrast with the actual psychoacoustical results (Figure 1B). Noticeably, the notch is still observed in the difference rate profiles at very high levels (upper panels in Figure 4B), provided that the notch is sufficiently deep (notch depth ≥ 9 dB). While at first sight this may seem inconsistent with the deterioration of the rate-profile representation of the notch due to the broadening of fibers' tuning, rate profiles are “noisy” and indeed the discrimination information available in the rate profile decreases gradually with increasing level beyond 80 dB SPL, as shown in the next section.

#### Population d′ estimates based on rate profiles are inconsistent with psychoacoustical threshold notch depth vs. level functions

The above conclusion was confirmed by a signal-detection-theory *d*-prime (*d*′) analysis of the physiological responses (Green and Swets, 1966; Shackleton et al., 2003). The “internal decision variable” in the psychoacoustical task was assumed to be proportional to the difference in firing rate between the flat-spectrum and notch conditions, assessed relative to the intrinsic variability in AN activity (the same stimulus token was used for all measurements for a given condition; hence, the variability in the responses arises exclusively from the stochastic nature of AN firing). A *d*′ for the population of AN fibers was calculated for all conditions as the square root of the sum of the squared-*d*′ values for individual AN fibers (Viemeister, 1988). This population *d*′ was compared with the psychoacoustical thresholds previously measured using a 3-alternative, forced-choice paradigm (Alves-Pinto and Lopez-Poveda, 2005). The relation between the AN population-*d*′ and the psychoacoustical threshold estimates did not need to be direct because, for example, as it is calculated, the population-*d*′ increases with the number of fibers in the sample. Nevertheless, the population-*d*′ provides a reasonable way of assessing, at least qualitatively, the expected perceptual performance based on intrinsically variable AN rate-profile information as a function of stimulus level.

**Figure 5:**
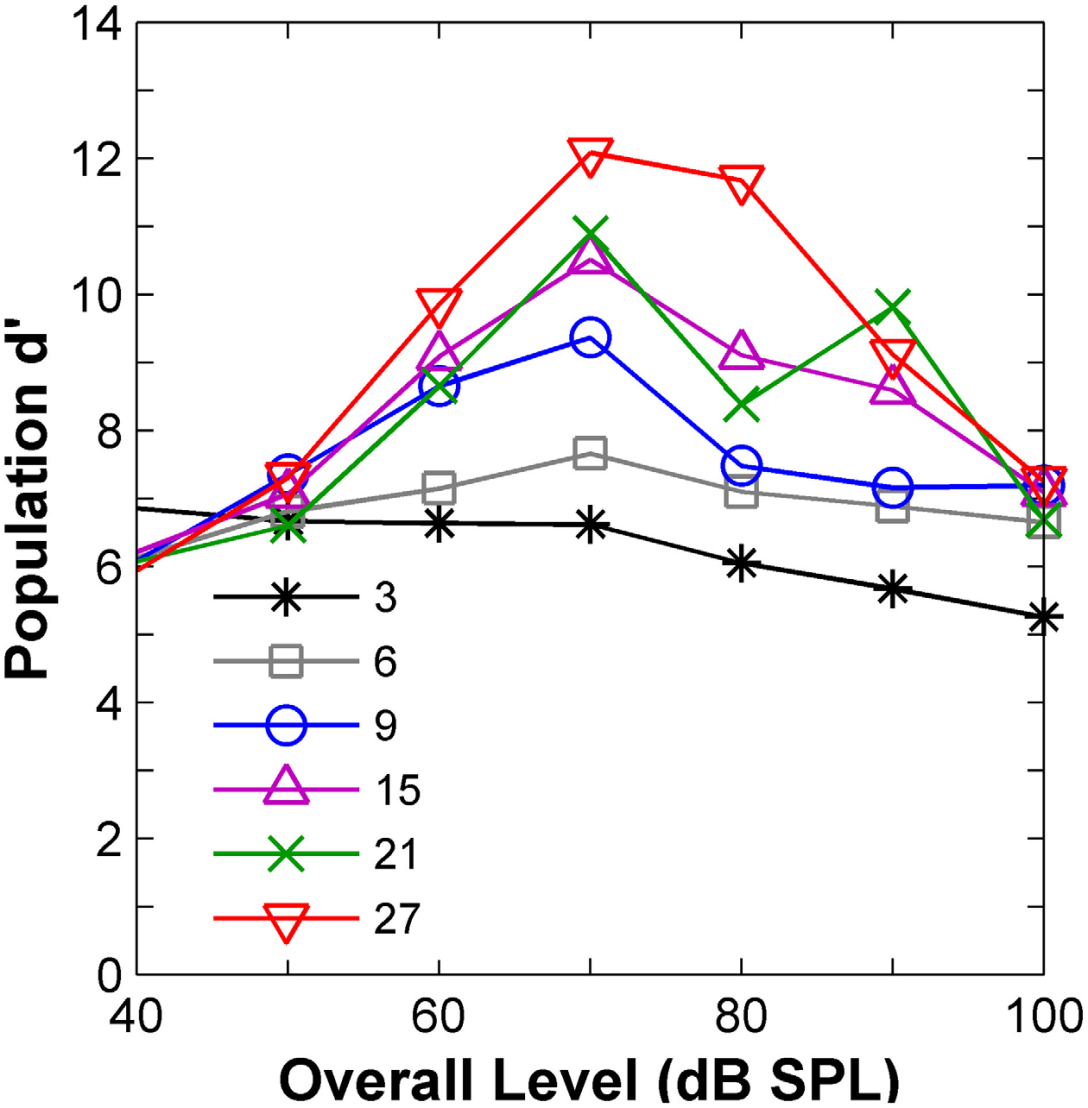
**Auditory nerve data: population *d*′ as a function of the noise overall level**. The numbers in the inset indicate notch depths in dB re spectrum level of the notch side bands.

The results (Figure 5) confirmed the insight gained from the visual analysis of the rate profiles (Figure 4) in terms of the effect of level. AN population-*d*′ values were highest (hence discrimination thresholds would be lowest), at levels around 70–80 dB SPL for virtually all notch depths (Figure 5), in clear contradiction with the perceptual results (Figure 1B), which suggested that *d*′ should be lowest around 80 dB SPL. In agreement with the evidence from the psychoacoustical and computer simulation studies (reviewed above), it can be, therefore, concluded that discrimination between auditory stimuli with different high-frequency spectral characteristics cannot be based on comparisons of their corresponding AN rate-profile representations.

#### Predicted performance based on the analysis of auditory nerve responses by an “ideal observer”

**Figure 6:**
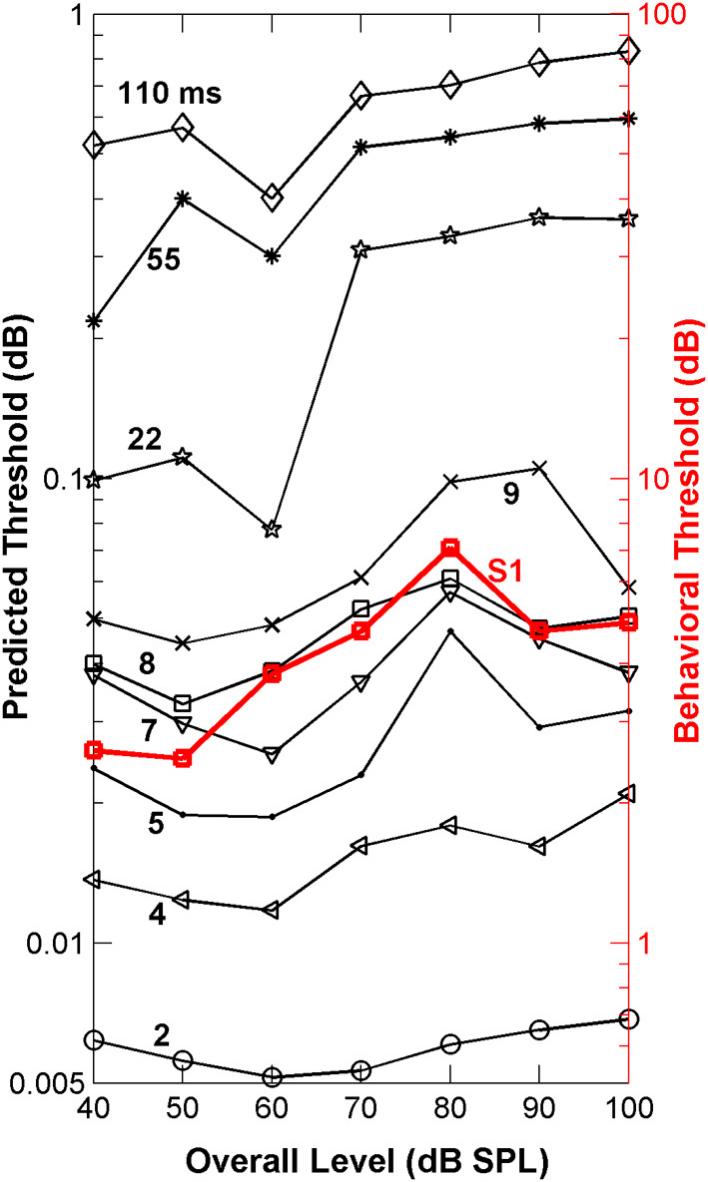
**Auditory nerve data: psychoacoustically observed notch-depth thresholds *vs*. “ideal observer” predictions from neural data**. Psychoacoustical (red squares, right ordinate axis) thresholds as a function of noise level for an example listener (S1; Figure 1B). Predicted thresholds (open symbols, left ordinate axis) were obtained using an “ideal observer” type of analysis of physiological AN responses [Equations (2a) and (2b)]. Different curves illustrate predicted thresholds when AN activity is analyzed over non-overlapping time binwidths of different durations, as indicated by the numbers next to each trace (in ms; adapted from Figure 1 of Lopez-Poveda et al., 2007).

The “ideal observer” analysis (Siebert, 1970; Heinz et al., 2001) is based on comparisons of discharge rates evoked by the two different stimuli (in this case a flat-spectrum noise and a noise with a 3-dB notch) computed in short non-overlapping time bins (Figure 3). This comparison between discharge rates was made for each single fiber and for each time bin of the fiber's PSTH (Figures 3A,B). Differences in discharge rate elicited by the two stimuli (filled *vs*. open bars in Figure 3A) vary across time bins with the sensitivity in each bin contributing additively to the overall sensitivity of each single fiber to the two stimuli (symbols in Figures 3A,B). By sensitivity we mean the ability of a fiber to discriminate between the flat-spectrum and the notch noises based on differences in discharge rate in each time bin elicited by the two stimuli [Equation (2b)]. This means that short-term differences in discharge rates evoked by the two stimuli, or equivalently, that temporal information, may also contribute discrimination information. Of course, different degrees of temporal information may be gained by sampling the instantaneous discharge rate in non-overlapping time bins of different durations; the shorter the time bin, the more precise the timing information, the greater the discrimination capability of the system, and the lower the discrimination thresholds. This was indeed found to be the case. For any given sound level, the predicted threshold notch depths decreased with shortening the sampling time bin (Figure 6). In absolute terms, however, the predicted thresholds were about two orders of magnitude lower than the behavioral ones (Figure 6). This mismatch likely reflects the pooling of information that occurs as different auditory inputs converge into higher nuclei in the auditory system. It may also reflect differences in cochlear processing between humans and guinea pigs, and/or that humans do not operate as optimal spectral discriminators, as others have suggested (Siebert, 1965, 1968, 1970; Delgutte, 1996; Heinz et al., 2001). Otherwise observed (psychoacoustical) and predicted (neural) absolute thresholds should match.

#### Monitoring nerve activity in shorter time bins of 4–9 ms predicted the level effect observed psychoacoustically

Remarkably, the *shape* of the predicted threshold notch depth *vs*. level functions varied greatly depending on the time binwidth. Only for time binwidths within the range from 4 to 9 ms were the predicted functions non-monotonic with a peak at or around 80 dB SPL, thus resembling the *shape* of most psychoacoustical functions (Figure 1B, and open red squares in Figure 6). This suggests that an effective cue for high-frequency spectral discrimination may be based on sampling rates of spike arrivals of AN fibers using non-overlapping time binwidths of between 4 and 9 ms (Figure 6).

**Figure 7:**
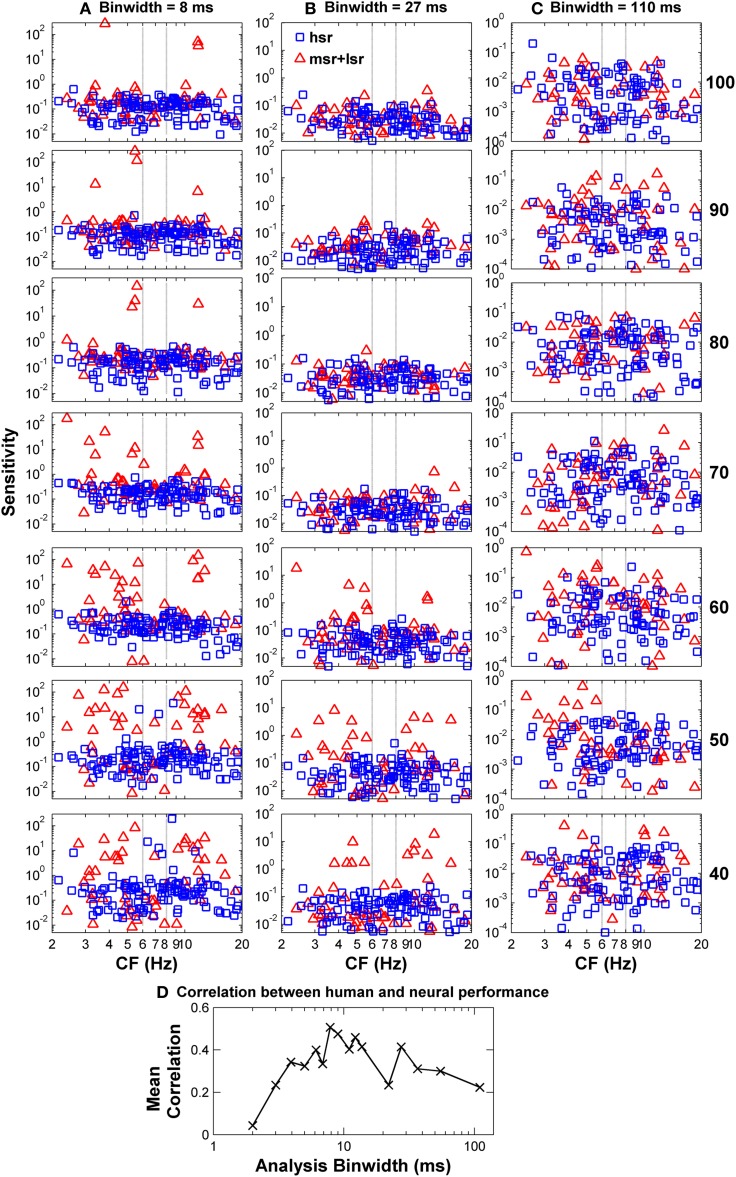
**Auditory nerve data: individual AN fiber sensitivity as a function of fiber's CF**. Sensitivity values for HSR and LSR fibers are illustrated by blue squares and red triangles, respectively. Sensitivity was calculated for three different time binwidths: 8 ms **(A)**, 27 ms **(B)**, and 110 ms **(C)**. Notice the different sensitivity scales used for the different binwidths. Stimulus level increases from the bottom to the top panel as indicated by the numbers on the right side of the figure (in units of dB SPL). Individual sensitivity values varied with stimulus level and with binwidth, with the highest sensitivity values occurring for different subgroups of fibers for different levels and binwidths. **(D)** Kendall's Tau non-parametric correlation between the *shape* of individual behavioral notch-depth thresholds (Figure 1B) and “ideal observer” neural predictions for different analysis time binwidths (black symbols in Figure 6). The figure illustrates the mean correlation coefficient values across five participants (Figure 1B).

To confirm this optimal analysis time binwidth, Kendall's τ non-parametric correlation coefficient (Press et al., 1992) was used to quantify the degree of correlation between the *shapes* of the predicted functions for different time binwidths and the observed functions for each one of five listeners (S1–S5, for which discrimination between flat-spectrum and a 2-kHz wide notch noise was tested) considered in the psychoacoustical study (Figure 1B). The actual degree of correlation varied considerably across listeners (not shown), but the highest correlations always occurred for a time binwidths between 7 and 9 ms. The mean value across subjects was approximately 8 ms (Figure 7D).

The notch depth threshold values predicted by the “ideal observer” analysis of AN fiber responses shown in Figure 6 were derived from the responses of the population of 163 AN fibers to the flat-spectrum and 3-dB notch noises. Analysis of individual fiber's sensitivity as a function of CF revealed that not all fibers contributed equally to the overall population sensitivity (Figure 7). Individual sensitivities for a binwidth of 8 ms showed that fibers with CF away from the notch band can contribute significantly to the population sensitivity (Figure 7A). Furthermore, the sub-populations of fibers with the highest sensitivities, therefore determinant to the discrimination threshold, also varied depending upon the analysis binwidth (compare Figures 7A–C).

The “ideal observer” analysis for a time binwidth equal to the stimulus duration (110 ms) disregards any temporal information. Hence, it was another way of testing the rate-profile code hypothesis. The shape of the associated predicted function (diamonds in Figure 6) clearly differed from that of the psychoacoustical function (red squares in Figure 6). Threshold notch depths were smallest for low-level sounds and gradually increased with increasing the sound level. Not surprisingly this shape resembles the curve that would be obtained by inverting the population-*d*′ *vs*. level function for a notch depth of 3 dB (Figure 5). Therefore, this analysis also indicates that the rate-profile is unlikely to provide the basis for high-frequency spectral discrimination.

#### Selective use of different fiber types does not account for the psychoacoustical discrimination as a function of level

The possibility exists that the non-monotonic shape of the behavioral threshold notch depth *vs*. level functions could reflect the existence of only two fiber types with different thresholds and dynamic ranges in the human AN, with the peak in the behavioral function occurring at the transition sound level between the dynamic ranges of the HSR and LSR fibers (Alves-Pinto and Lopez-Poveda, 2005). This mechanism has been put forward as one way that the AN handles information over a much wider range of sound levels than the dynamic range of its individual fibers; that is, as a solution for the dynamic range problem of hearing (Viemeister, 1988; Delgutte, 1996).

This conjecture was tested here by applying the “ideal observer” analysis to two groups of AN fibers, with units classified according to spontaneous rate as HSR or LSR+MSR when their spontaneous rate was higher or lower than 18 spikes/s, respectively (Liberman, 1978). The resulting HSR and LSR+MSR groups contained 110 and 53 fibers, respectively. The mean optimal time binwidth of 8 ms (Figure 7D) was used.

**Figure 8:**
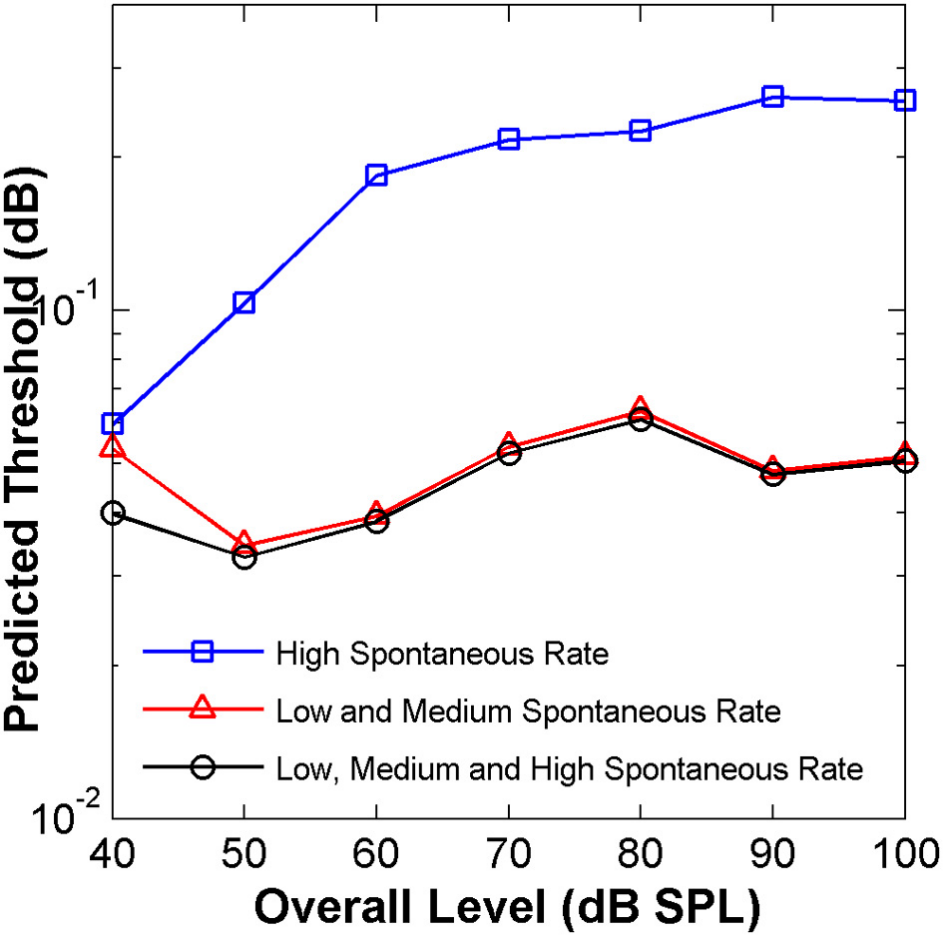
**Auditory nerve data: predicted threshold notch depth *vs*. level functions from an “ideal observer” analysis of neural responses from different fiber types, as indicated in the legend**. Predictions are for an analysis time window of 8 ms.

Predicted threshold notch depth *vs*. level functions differed for the two groups (Figure 8). Nevertheless, predicted thresholds at low sound levels were lower for the LSR+MSR group than for the HSR group. This means that LSR+MSR fibers are more sensitive to spectral changes at low sound levels than are HSR fibers. Most important is, perhaps, that the predicted functions were almost identical for the LSR+MSR group and for the combined HSR+LSR+MSR sample, and that both their shapes were highly correlated with the shape of the perceptual discrimination functions (Figure 1B). This suggests that LSR+MSR fibers may be more significant to high-frequency spectral discrimination than are HSR fibers at all sound levels tested. This result indicates that the non-monotonic shape of the behavioral discrimination functions is unlikely to reflect a transition between the dynamic ranges of the two fiber types.

#### The effect of stimulus duration

In the psychoacoustical discrimination study, it was observed that threshold notch depths for discrimination were on average 2.5 times larger for a short (20-ms duration) than for a long (220 ms) stimulus, and that this ratio was approximately constant across sound levels (Alves-Pinto and Lopez-Poveda, 2005). In other words, the effect of level was independent of stimulus duration. The ideal observer analysis was therefore used to predict the behavioral thresholds for stimulus durations of 110 and 20 ms. A time binwidth of 5 ms was used in this case for convenience because it is a submultiple of these two stimulus durations.

**Figure 9:**
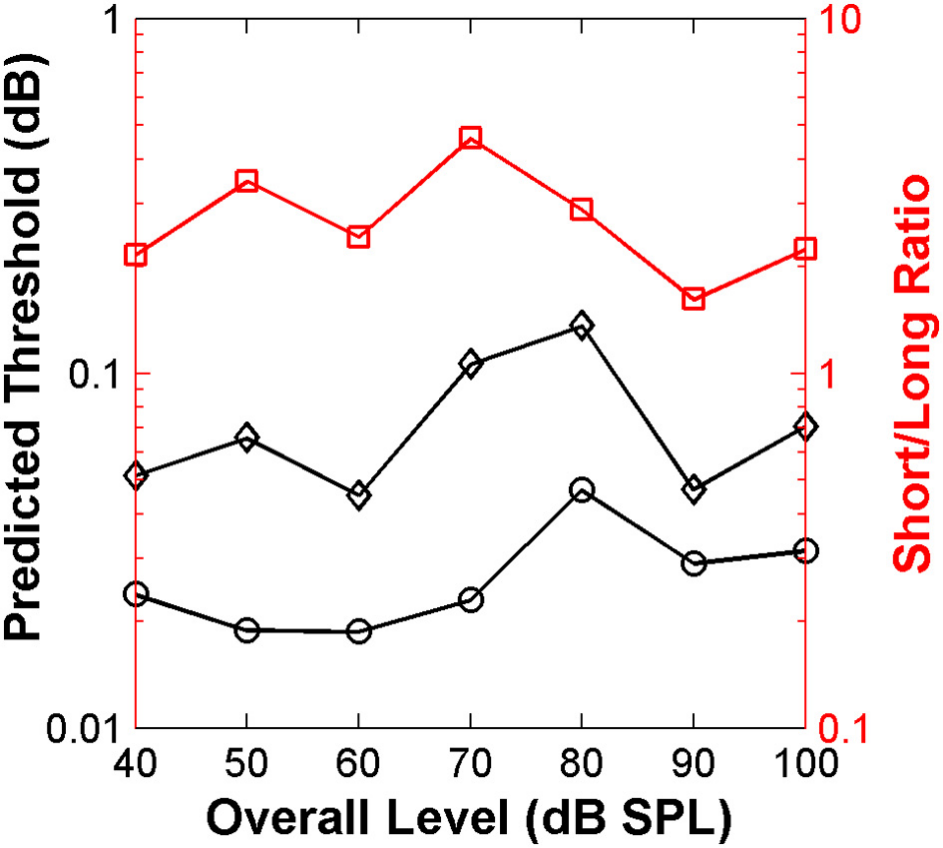
**Auditory nerve data: threshold notch depth *vs*. level functions predicted by an “ideal-observer” analysis of neural responses for noise bursts with different duration: 20 ms (diamonds) and 110 ms (circles)**. Red squares (right ordinate axis) illustrate the ratio between predicted thresholds for the long and short stimuli.

The resulting predicted thresholds were higher for the short than for the long stimulus (Figure 9). Moreover, the ratio between the two values (red squares in Figure 9) was similar across levels and on average equal to 2.8. These results match well with those from the main psychoacoustical study (Alves-Pinto and Lopez-Poveda, 2005). This match reveals that the “ideal observer” analysis provides a reasonable account of the behavioral discrimination thresholds based on the *relative* neural information available for the short and long stimuli. In the context of the present analysis, we would suggest that higher thresholds resulted from having fewer time bins in which to assess differences between the neural responses to the two stimuli.

### Discussion of experimental neural findings

We have shown that psychoacoustical discrimination between auditory broadband stimuli with and without high-frequency spectral notches is uncorrelated with the differences in the overall AN rate-profile representations of their spectra. Although the spectral notch is visible in the rate-profile for all sound levels above 50 dB SPL provided it is sufficiently deep (Figure 4B), the effect of level on the quality of that neuronal representation does not match, and therefore is unlikely to explain, the effect of level in the behavioral notch discrimination thresholds. Altogether, the present neural results are inconsistent with the view that high-frequency spectral features are encoded in the AN average-rate profile (e.g., Rice et al., 1995), and support the inferences made from the related human masking patterns (Figure 1C) and computational modeling studies (Figure 2). The present AN results support a combined rate-time code instead. The nature of the code is uncertain, but the present analysis suggests that information decoding requires sampling the discharge rate of the fiber population in time binwidths of approximately between 4 and 9 ms. Unfortunately the number of stimulus repeats used here for the physiological experiments was insufficient to draw reliable conclusions and further experimental evidence is still necessary to confirm the present conclusions, to dismiss the rate profile as the only encoding strategy for high frequency features, and to elucidate the nature of the rate-time code underlying high-frequency spectral discrimination.

Differences in neuronal processing between humans and guinea pigs may have contributed to the mismatch between the psychoacoustical and the neural results in terms of level dependence of rate-profile derived discrimination thresholds. Also the anesthetic may have had an effect on neuronal responses. Both of these factors would have however also affected the correspondence between psychoacoustic and neural results based on the “ideal-observer” analysis. Nevertheless, the idea that some form of temporal code may be used for high-frequency spectral discrimination is not new and agrees with evidence from other independent studies in a number of aspects. It has been put forward, for example, to explain the limits of human auditory frequency discrimination for single tones (Heinz et al., 2001) and for the sensitivity to the spectral fine-structure of sounds in the high-frequency range (> 4 kHz; e.g., Moore and Sek, 2009). The results presented here support this principle. Furthermore, the present neural results extend the validity of the principle to spectral discrimination of broadband *aperiodic* stimuli (which is a more natural type of auditory task than pure tone discrimination) and reveal the existence of an optimal decoding time binwidth of 8 ms.

What is the nature of the temporal code? We have no definite answer, only conjectures. Any AN fiber is effectively driven by a half-wave rectified, low-pass filtered version of the basilar membrane response waveform at its corresponding place in the cochlea. With broadband noise stimulation, this response can be described as a randomly amplitude-modulated carrier with a carrier frequency near the fiber's CF. The range of modulation frequencies is limited by the BW of the cochlear filter (Louage et al., 2004) or the cut-off of phase locking. The BW of basilar membrane responses increases with increasing sound level (Robles and Ruggero, 2001). Therefore, the range of modulation frequencies as well as the phase of the basilar membrane response waveform both depend on sound level. AN fibers can phase-lock to the envelope of basilar membrane excitation even at high levels, when their discharge rate is at saturation (Cooper et al., 1993). Given that fibers with CFs near the notch frequency surely “see” a different level than those with CFs well away from it, it is therefore, possible that spectral discrimination be based on detecting either the range of modulation frequencies or the phase differences implicit in AN spike trains (or both). In other words, the auditory system might be treating a spectral discrimination task as an envelope discrimination task; the envelope being that of the signals coming from different cochlear channels. An envelope-based discrimination code would be consistent with the found optimal time binwidth of 4–9 ms.

That said, however, any difference in the envelopes evoked by the flat-spectrum and notch noises should show up in the aggregated FFTs of the simulated IHC receptor potential waveforms; that is, they should show up in Figures 2C,D. Admittedly, some differences between the FFTs for the two noises did indeed occur for frequencies below 100 Hz (not shown in Figures 2C or 2D) but they were almost negligible and much smaller than the differences in the notch frequency band highlighted in Figure 2D. Insofar as Figure 2C represents an upper limit to the periodicities that can be represented via phase locking in the AN-fiber population by the “volley principle” (Wever, 1949), Figure 2C suggests that the fine-time structure of AN activity would be a stronger cue for high-frequency spectral notch discrimination than the information available through synchronized responses to the envelopes. Unfortunately, gathering spectral information from the timings of spikes for spectral components around 7 kHz would require analyzing spike trains with very short binwidths, of 0.14 ms, and to avoid artifactual results [i.e., very high sensitivity due to close-to-zero discharge rate, Equations (1) and (2b)], this would require having many more repeats for each fiber than we have measured. For this reason, we could not confirm or reject this hypothesis using the available data. In summary, further experimental evidence is still necessary to clarify the nature of the temporal code.

The present neural results support the “multiple-looks” model for auditory long-term temporal integration: the decrease in threshold with increases in the stimulus duration. Such temporal integration does not actually involve integrating stimulus energy (or correspondingly accumulating nerve spikes) over time, but is more consistent with a model whereby “multiple-looks” of the output envelopes from auditory filters are taken in non-overlapping time windows of about 5–10 ms of duration (Viemeister and Wakefield, 1991). The “looks” would be stored in memory and accessed selectively for further processing and decision making. This model was proposed to account for behavioral observations, but has lacked physiological support to date. The present physiological results are consistent with such a model and even the range of optimal time binwidths found here (4–9 ms) matches the duration of the time windows proposed in the “multiple-looks” model.

The present physiological results are also consistent with explanations proposed for the so-called “dynamic range problem” of hearing. This refers to the apparent mismatch between the wide range of sound levels over which good intensity discrimination can be shown and the dynamic range of most AN fibers (Viemeister, 1988; Delgutte, 1996; Moore, 2003). Several different mechanisms are likely to contribute, but none of them seems to be critical or to fully explain the various behavioral results (Delgutte, 1996). Some models indicate that an appropriate combination of information from only a *few* AN fibers can account for intensity discrimination thresholds, even at high intensities (Delgutte, 1987; Viemeister, 1988). Further, they indicate that the activity of LSR fibers determines behavioral performance at high sound levels (Viemeister, 1988). The present study concerns a different perceptual task, but the results provide experimental support to those ideas. Here it was observed that only a handful of highly-sensitive fibers sufficed to produce the observed improvement in discrimination at very high sound levels (> 80 dB SPL) (Figures 1B, 6). Furthermore, the subpopulation of LSR+MSR fibers appears to convey enough information to account for most of the psychoacoustical thresholds (Figures 7, 8). Interestingly, this was true over the whole range of sound levels that were used.

Some questions remain. First, the “ideal observer” predictions showed that performance could improve substantially if the discharge rate of AN fibers were sampled in time binwidths shorter than 8 ms (Figure 6). This is true even allowing for the fact that humans do not operate as optimal discriminators, hence the two-order-of-magnitude difference between psychoacoustical and predicted thresholds. That is, it seems as though humans are not using all the information available in the AN. On the other hand, the value of 8-ms for the optimal time binwidth does not seem coincidental. It matches well with the conclusions from the “multiple-looks” model. Furthermore, there is also indirect evidence that visual information is processed in time windows of comparable durations (Van Rullen and Thorpe, 2001). The question is what does it mean? One possibility is that it relates to the time constant of cochlear nucleus neurons specialized in spectral-notch or spectral-edge detection (Reiss et al., 1995; Zheng and Voigt, 2006).

Second, the amount of perceptually-relevant information for high-frequency spectral discrimination was shown to be less for sound levels around 80 dB SPL than for lower or higher levels. This still needs explaining. The results presented here demonstrate that it is unrelated to having two fiber populations with different thresholds and dynamic ranges. It is possible that spectral representation of the notch in the BM excitation pattern may be compromised at mid-levels due to cochlear mechanical compression (see Lopez-Poveda et al., 2008).

## Potential implications for understanding across-listener variability in sound localization

### Spectral-notch cues vary across listeners

It has been long thought that high-frequency spectral notches in the head-related transfer function (HRTF) are important cues for human (vertical) sound localization (e.g., Butler and Belendiuk, 1977; Butler and Humanski, 1992). On the other hand, the depth and the BW of HRTF notches vary widely across listeners [(see, for instance, Shaw (1982) or Chapter 3 in Lopez-Poveda, 1996)], probably reflecting differences in ears' shape and size across listeners (Lopez-Poveda and Meddis, 1996). Furthermore, we have shown that notch depth at discrimination threshold varies widely across listeners (Figure 1B) and depends on the notch BW as well on stimulus level and duration (Alves-Pinto and Lopez-Poveda, 2005). Assuming that behavioral discrimination between flat-spectrum and notch noises is based on the quality of the internal representation of the notches, then, in light of the present evidence, sound localization accuracy should vary across listeners, should be more precise for long than for short stimuli and for levels below 60–70 dB SPL than for levels around 70–80 dB SPL and this is indeed the case (Hartmann and Rakerd, 1993; Macpherson and Middlebrooks, 2000; Vliegen and Van Opstal, 2004; Macpherson and Sabin, 2013). Furthermore, vertical localization accuracy should improve for levels higher than about 80 dB SPL, although this remains to be tested.

In any case, the ability of listeners to actually use high-frequency HRTF notches as sound localization cues must depend on a complex combination of their level of performance in notch detection tasks, the shape of their ears, and the characteristics of the stimulus (duration and level).

### Potential variability associated to neural encoding of spectral features

Performance in high-frequency notch detection tasks, and hence in spatial localization involving detection of these spectral features, will ultimately depend on the quality of the representation of the spectral notch in the AN. The evidence provided here suggests that high-frequency spectral information may be encoded in the temporal pattern of AN discharges, analyzed over time binwidths 4–9 ms long. Studies on the temporal aspects of spectral processing in sound localization also reported that information about the spectrum level of a cochlear filter can only be reliably obtained when the signal from that filter is integrated over a time window of about 5 ms (Jin, 2001), a duration similar to that estimated from the “ideal observer” analysis of AN fibers' responses (Figure 7D).

Spectral notch encoding based on the temporal patterns of discharge of AN fibers is likely to be more susceptible to variability than encoding based on the long-term average discharge rate. Spikes occur stochastically in time and spike counts for constant stimuli are likely to vary from time bin to time bin. Variations in the number of spikes have a larger effect in a small than in a larger time window, making any changes that are not stimulus related to more strongly affect the quality of the information encoded in the spike pattern. This higher susceptibility to variability could partly contribute to the large variability in the detection of spectral notches across listeners observed here.

Finally, discrimination thresholds derived from the “ideal observer” analysis of responses of LSR and MSR fibers were comparable to those derived using all fibers, including HSR fibers (Figure 8). This suggests that LSR and MSR fibers, despite their being a smaller population, are more sensitive to high-frequency spectral differences than are HSR fibers at all levels and so that LSR and MSR fibers could be key for detecting high-frequency spectral notches. Furthermore, it suggests that high-frequency notch discrimination would be probably impaired by damage and/or loss of these more sensitive fibers. According to a recent report (Furman et al., 2013), noise exposure selectively damages LSR fibers without altering audiometric thresholds. It has been suggested that this significantly impairs hearing in noise (Lopez-Poveda and Barrios, 2013). It is possible, therefore, that different audiometrically normal listeners may suffer from different degrees of (hidden) LSR fiber loss, depending on their individual histories of noise exposure and/or genetic sensitivity to noise, which would lead to variable performance in spectral discrimination tasks and, consequently, to variable performance in spatial localization involving the detection of high-frequency spectral notches. Further research is required to test this conjecture.

## Conclusions

For most listeners, high-frequency spectral notch detection becomes gradually more difficult with increasing level up to 70–80 dB SPL and improves at higher levels. However, across-listener variability is high and depends both on the stimulus characteristics (duration and level) and on the notch BW.

Psychoacoustical, modeling, and physiological results consistently suggest that the non-monotonic effect of level on notch detection is inconsistent with the notch being encoded in the rate profile of AN fibers only and support, instead, that the temporal pattern of AN discharges monitored in time binwidths of 4–9 ms of duration conveys encoding relevant information. Physiological data suggest that LSR fibers are key to notch encoding.

The present evidence suggests that high-frequency spectral notch detection, and consequently, also vertical sound localization accuracy, requires information carried in the temporal characteristics of AN activity, particularly, by the available number of low and medium spontaneous rate fibers. The number of fibers likely varies substantially across individuals, which might contribute to across-listener variability in sound localization.

### Conflict of interest statement

The authors declare that the research was conducted in the absence of any commercial or financial relationships that could be construed as a potential conflict of interest.

## Footnotes

^1^90–95% of the population of spiral ganglion neurons comprise type I cells. These are connected to the inner hair cells and encode most auditory information. The rest 5–10% of the population consist of type II afferents that are connected to outer hair cells. Their role in auditory coding remains unclear but they are likely involved in the regulation of the operating point of the “cochlear amplifier” (Pickles, 1988; Jagger and Housley, 2003; Ashmore et al., 2010).
